# Diabetic Peripheral Neuropathy in Ambulatory Patients with Type 2 Diabetes in a General Hospital in a Middle Income Country: A Cross-Sectional Study

**DOI:** 10.1371/journal.pone.0095403

**Published:** 2014-05-01

**Authors:** María de los Angeles Lazo, Antonio Bernabé-Ortiz, Miguel E. Pinto, Ray Ticse, German Malaga, Katherine Sacksteder, J. Jaime Miranda, Robert H. Gilman

**Affiliations:** 1 CRONICAS Centre of Excellence in Chronic Diseases, Universidad Peruana Cayetano Heredia, Lima, Peru; 2 Unidad de Conocimiento y Evidencia, Universidad Peruana Cayetano Heredia, Lima, Peru; 3 Facultad de Medicina “Alberto Hurtado”, Universidad Peruana Cayetano Heredia, Lima, Peru; 4 Servicio de Endocrinología, Hospital Nacional Cayetano Heredia, Lima, Peru; 5 Department of International Health, Johns Hopkins Bloomberg School of Public Health, Baltimore, Maryland, United States of America; University of Florida, United States of America

## Abstract

**Aim:**

We aimed to estimate the morbidity rate and associated factors for diabetic peripheral neuropathy (DPN) in a low-middle income country setting.

**Methods:**

Cross-sectional study, data was gathered at Peru's Ministry of Health national specialized hospital for endocrinological conditions through standardized interviews, anthropometric measurements and blood tests for glycated haemoglobin (HbA1c). DPN was evaluated using two techniques: the Semmes-Weinstein monofilament test and the diabetic neuropathy symptom score. Overall prevalence and 95% confidence intervals (95% CI) were calculated. Potential factors related to DPN explored included body mass index, years with disease (<10 vs. ≥10 years), glycaemic control (HbA1c <7% vs. ≥7%), microalbuminuria, retinopathy, and current pharmacological treatment. Multivariable analysis was performed using Poisson analysis to calculate prevalence ratios.

**Results:**

DPN was observed in 73/129 (56.6%) patients. In multivariable analysis adjusted by age and sex, the prevalence ratio of neuropathy was 1.4 times higher (95% CI 1.07–1.88) in patients who took insulin plus metformin compared to patients who used one treatment alone, and 1.4 higher (95% CI 1.02–1.93) in patients with ≥10 years of disease compared to those with a shorter duration of disease. Also we found some characteristics in foot evaluation associated to neuropathy such as deformities (p<0.001), onychomycosis (p = 0.012), abnormal Achilles reflex (p<0.001), pain perception (p<0.001) and vibration perception (p<0.001).

**Conclusion:**

DPN is highly frequent among patients with diabetes in a national specialized facility from Peru. Associated factors to DPN included being a diabetic patient for over ten years, and receiving insulin plus metformin

## Introduction

Type-2 diabetes mellitus (T2DM) constitutes a major challenge in low-middle income countries (LMIC): only 3.6% of patients in Latin America achieve the three American Diabetes Association (ADA) recommended targets for blood pressure, LDL cholesterol, and HbA1C [1]. This demonstrates that a significant gap between recommended diabetes care and the care patients actually receive exists in LMIC.

Diabetic foot neuropathy is one of the most important complications and can have a huge impact on patients, families, and society. Cross-sectional studies from LMIC found prevalence rates of DPN between 22%–60% [2], [3], and up to 25% of them will develop a foot ulcer [4]. Eighty-five percent of lower-limb amputations are preceded by foot ulceration [5], and 5-year mortality following amputation is 44.3%[6]. Improvements in foot ulceration diagnosis and prevention can reduce amputation risk and reduce health care costs [7]. Prior to assessing appropriate methods to diagnose and prevent these complications, it is necessary to better understand the prevalence and characterize associated risk factors in our population. Therefore, the primary objective of the study was to determine the prevalence of DPN in ambulatory patients in a general national hospital in an LMIC setting and the secondary objective was to explore associated factors for DPN in this population.

## Methods

### Ethics Statement

Hospital Nacional Cayetano Heredia's (HNCH) Ethics Committee approved the study protocol and written informed consent was obtained from all participants.

### Study Design and participants

We conducted a cross-sectional study between July and August 2012 at the diabetes outpatient clinic of HNCH in Lima, Peru. Recruitment strategy was convenience sampling strategy of patients receiving care in this facility, selected by Peru's Ministry of Health as the national specialized hospital for endocrinological conditions [8]. Inclusion criteria were T2DM diagnosis according to ADA criteria (HbA1c ≥6.5% or Fasting plasma glucose ≥126 mg/dl or two-hour plasma glucose ≥200 mg/dl during an oral glucose tolerance test or a random plasma glucose ≥200 mg/dl) and age ≥18 years old. Patients with gestational diabetes mellitus and secondary diabetes (steroid-induced, cystic fibrosis, hemochromatosis and chronic pancreatitis) were excluded [9].

### Assessments and outcome measures

Venous blood samples were evaluated for glycated haemoglobin (HbA1c) using high-performance liquid chromatography (D10-BIORAD, Germany). According to ADA guidelines, glycaemic control was defined as HbA1c <7.0% and blood pressure control as <140/80 mmHg [10].

Potential factors related to DPN explored included body mass index, years with disease (<10/≥10 years), glycaemic control (HbA1c <7%/≥7%), microalbuminuria, retinopathy, and current pharmacological treatment. Drug treatment was categorized into oral hypoglycaemic agents (OHA), insulin, and OHA plus insulin. Urine samples were assessed for microalbuminuria using commercial test strips following manufacturer's instructions: albumin concentrations ≥20 mg/L are consistent with microalbuminuria (albumin >30 mg/day). Retinopathy was evaluated through a fundoscopy exam by an ophthalmologist.

We completed a checklist to assess characteristics of the appearance of the feet, noting deformities, dry skin, calluses, muscle wasting (guttering between metatarsals), onychogryphosis (hypertrophic nails), onychomycosis, infection, and cracks. We also performed vascular and neurological evaluations. For the vascular evaluation, palpation of the dorsalis pedis pulse was performed using fingertips and the presence or absence was recorded. Sensory neurological evaluation consisted of: (i) ankle jerk (Achilles reflex), (ii) pain perception using a sterile pin in the dorsum of the great toe, and (iii) vibration with a 128-Hz tuning fork.

Additionally, we performed the Semmes-Weinstein monofilament (SWF) test and the 4-item diabetic neuropathy symptom (DNS) score. Nylon monofilaments were constructed to buckle when a 10-g force was applied, and loss of the ability to detect this pressure on the plantar surface of the foot indicated loss of large-fibre nerve function. Two sites of the foot, first metatarsal heads and plantar area of the heel were examined [11]. Inability to feel a SWF was considered to be indicative of high risk for foot ulceration [12]. The DNS score was validated by Meijer *et al*. [13] and consists of the following self-reported items: (1) unsteadiness during walking, (2) pain, burning or aching of the legs or feet, (3) prickling sensations in the legs or feet, and (4) numbness in the legs or feet. Presence is scored as 1 point, absence as 0 points, with a maximum score of 4 points. A DNS score of ≥1 point is considered positive. The same physician performed all of the SWF and DNS tests in the same patient. DPN was defined as a negative SWF test at one point and/or a DNS score between 1 and 4.

### Statistical methods

The calculated sample size was 114, which assumed a 40% prevalence of neuropathy, [14] 0.09 precision and 0.05 significance. Prevalence and 95% confidence intervals (95% CI) were calculated. Chi-square or Fisher's exact test, where appropriate, were used to explore potential factors related to DPN. Multivariable analysis was performed using Poisson analysis for calculating prevalence ratios. STATA 11 for Windows (Stata Corp, College Station, TX) was used for analysis.

## Results

A total of 129 T2DM patients, 73 (56.6%) females, mean age 59.2 years (SD ±8.6), were enrolled in the study. Mean body mass index was 28.9 Kg/m^2^ (SD ±5.2), mean duration of T2DM was 8.6 years (SD ±6.5) and mean HbA1c was 8.7% (SD ±2.2). Mean systolic and diastolic blood pressure were 121.5 mm Hg (SD ±22.3) and 70.9 mm Hg (SD ±11.0), respectively. Sixty-two patients (48.0%) took OHA as treatment for their diabetes, while 12 (9.3%) took insulin alone, and 28 (21.7%) took OHA + insulin.

DPN was present in 73 (56.6%; 95% CI 47.9%–65.2%) patients. Of these, 49 (37.9%) patients had a positive DNS score and 51 (39.5%) patients had a negative response to SWF; 27 (20.9%) patients were positive on both tests.

Only 26.4% (95% CI 18.6%–34.1%) of the study population met the ADA glycaemic goal, while 71.3% (95% CI 62.7%–78.9%) met the ADA blood pressure goal. Forty-two (32.5%; 95% CI 24.3%–40.7%) patients took anti hypertensive drugs. Characteristics of the population with and without neuropathy are presented in Table 1. Table 2 shows the results of the evaluation of the appearance of the feet, the vascular evaluation, and the neurological assessment with respect to DPN.

**Table 1:** Characteristics of the study population according to neuropathy.

	Neuropathy (N = 73)	Without neuropathy (N = 56)	p-value
Sex			
** Female**	42 (57.5%)	31 (42.5%)	0.80
** Male**	31 (55.3%)	25 (44. 6%)	
Age			
** <60 years**	33 (50.7%)	32 (49.2%)	0.17
** ≥60 years**	40 (62.5%)	24 (37.5%)	
Body Mass Index			
** <25**	12 (57.1%)	9 (42.9%)	0.66
** ≥25 and <30**	31 (52.5%)	28(47.5%)	
** ≥30**	30 (61.2%)	19 (38.85%)	
Years with disease			
** <10 years**	31 (45.6%)	37 (54.4%)	0.008
** ≥10 years**	42 (68.8%)	19 (31.2%)	
Glycated haemoglobin			
** <7%**	17 (50%)	17 (50%)	0.36
** ≥7%**	56 (58.6%)	39 (41.1%)	
MIcroalbuminuria			
** Negative**	57 (56.4%)	44 (43.5%)	0.94
** Positive**	16 (57.1%)	12 (42.8%)	
Retinopathy			
** Yes**	19 (73.1%)	7 (26.9%)	0.048
** No**	52 (51.4%)	49 (48.5%)	
Treatment			
** Any other treatment**	51 (50.5%)	50 (49.5%)	0.008
** OHA** ** **+ insulin**	22 (78.6%)	6 (21.4%)	

*Percentages were calculated by rows.

**OHA: Oral hypoglycaemic agent.

**Table 2:** Characteristics of foot evaluation.

	Neuropathy (N = 73)	Without neuropathy (N = 56)	
Appearance of feet			
** Deformities**	27(37.0%)	4 (7.14%)	<0.001*
** Drew skin, calluses**	68(93.2%)	52 (92.8%)	0.948
** Hypotrophy interosseous**	38(52.1%)	19 (33.9%)	0.05
** Onychogryphosis**	26(35.6%)	13 (23.2%)	0.12
** Onychomycosis**	62 (84.9%)	36 (54.2%)	0.012*
** Infection**	1 (1.37%)	-	0.37
** Cracks**	25 (34.2%)	13 (22.1%)	0.17
Vascular evaluation			
** Dorsalis pedis pulse absent**	2 (2.7%)	2 (3.6%)	0.78
Neurological evaluation			
**Achilles Reflex abnormal**	63 (86.3%)	20 (35.7%)	<0.001*
**Pain perception abnormal**	46 (63.1%)	6 (10.7%)	<0.001*
**Vibration perception abnormal**	32 (43.8%)	7 (12.5%)	<0.001*
Ulceration	1 (1.37%)	1 (1.79%)	

*Fisher exact test.

In the multivariable model, years of disease and treatment with OHA plus insulin were factors associated with increased prevalence of DPN (Table 3).

**Table 3:** Factors associated with Diabetic Peripheral Neuropathy.

	Bivariable model Prevalence ratio (95% CI)	Multivariable model* Prevalence ratio (95% CI)
Sex		
** Female**	1 (Reference)	
** Male**	0.96 (0.71–1.31)	
Age		
** <60 years**	1 (Reference)	
** ≥60 years**	1.23 (0.91–1.67)	
Body Mass Index		
** <25**	1 (Reference)	
** ≥25 and <30**	0.92 (0.59–1.43)	
** ≥30**	1.07 (0.69–1.65)	
Years with disease		
** <10 years**	1 (Reference)	1 (Reference)
** ≥10 years**	1.51 (1.11–2.06)	1.41 (1.03–1.93)
Glycated haemoglobin		
** <7%**	1 (Reference)	
** ≥7%**	1.18 (0.81–1.72)	
MIcroalbuminuria		
** Negative**	1 (Reference)	
** Positive**	1.01 (0.70–1.46)	
Retinopathy		
** No**	1 (Reference)	
** Yes**	1.42 (1.05–1.92)	
Treatment		
** Any other treatment**	1 (Reference)	1 (Reference)
** Metformin + insulin**	1.56 (1.18–2.05)	1.43 (1.08–1.89)

*Variables independently associated with DPN.

## Discussion

We found that DPN is highly frequent among patients with T2DM in a national specialized facility from Peru. According to the literature, DPN prevalence varies from 10% to 75% [15]–[17], a wide range that reflects differences among countries, settings, diagnostic methods, population characteristics, and quality of care. Only two studies have presented data on DPN prevalence in Latin American countries. One study in an outpatient clinic in Brazil found a prevalence of 22%–46% depending on method used—nerve conduction and the Michigan questionnaire, respectively [2]. Another study in Mexico found a prevalence of 69% when evaluating DPN by the Michigan questionnaire [18]. The lack of data on DPN in Latin American countries underscores the need for further investigations of its epidemiology.

In our study, more than half of the T2DM patients had peripheral neuropathy when evaluated by the DNS score and SWF test, both techniques have high sensitivity, a characteristic that is ideal for screening. However, we did not use the gold standard of nerve conduction, and thus we may have overestimated the prevalence of DPN within this sample. Nerve conduction is too expensive for regular use in LMIC, and it is important to investigate alternative cheaper diagnostic approaches. However, future studies should perform more extensive comparisons of DPN in Latin American countries using a standard method.

Most previous studies found that duration of diabetes, insulin treatment, proteinuria, and presence of retinopathy are factors associated with DPN [19], [20], and our results are consistent with these findings. Drug treatment is an important risk factor for neuropathy, which may be related to the duration of disease. In one cohort study, patients receiving insulin monotherapy and insulin plus metformin were twice as likely to develop neuropathy when compared to patients taking metformin alone [21]. Similarly, we found that patients taking insulin plus OHA were 40% more likely to have DPN, as were those with more than 10 years of disease. This finding clearly shows that patients with severe disease, that need more than one treatment scheme are most likely to have neuropathy.

Our work in Peru reveals a very low prevalence of glycaemic control among patients with T2DM. Previous studies demonstrate that foot deformities and other ulcer risk factors are exacerbated by poor glucose control and longer duration of diabetes [22]. Similarly, the prevalence of complications such as foot ulcers is significantly higher in patients with higher HbA1c levels [23]. Together with the associated chronic complications, this constitutes a major health concern in LMIC such as Peru.

A major limitation of our study is the selection of patients with T2DM from a single facility. However, according to Peru's Ministry of Health, this study site has the status of leading national specialized hospital for endocrinological conditions [8]. Thus, characterizing DPN in this hospital is of enormous importance for establishing bench marks in the care patients with diabetes in Peru as well as to guide future policy and quality improvement developments.

Based on our findings, we can expect to find high rates of long-term diabetes-related complications in Peru if control strategies are not implemented. While our results are not necessarily directly translatable to other centres, they emphasize the need for developing monitoring and evaluation approaches that are appropriate in resource-constrained settings. Screening of high-risk individuals, early detection, and proper management of DPN in patients with T2DM should be mandatory. As per guidelines, careful foot examination at each visit to the outpatient clinic, active application of screening tools every year, and an organized diabetic foot care program should be implemented to prevent complications in patients with T2DM
